# Myocarditis in Mediterranean spotted fever: a case report and a review of the literature

**DOI:** 10.1099/jmmcr.0.005039

**Published:** 2016-08-30

**Authors:** Claudia Colomba, Lucia Siracusa, Marcello Trizzino, Claudia Gioè, Anna Giammanco, Antonio Cascio

**Affiliations:** Dipartimento di Scienze per la promozione della salute e materno-infantile, Università di Palermo, Italy

**Keywords:** myocarditis, rickettsia, conorii, Mediterranean, spotted, fever

## Abstract

**Introduction:**

Mediterranean spotted fever (MSF) is a tick-borne acute febrile disease caused by *Rickettsia conorii*. Most cases follow a benign course, with a case fatality rate of 3–7 % among hospitalized patients. Complications are described mainly in adult patients and include hepatic, renal, neurological and cardiac impairment. Among cardiac complications, pericarditis, myocarditis and heart rhythm disorders are uncommon complications in MSF and only a few cases have been reported in the literature.

**Case Presentation:**

We describe a new case of acute myocarditis complicating MSF in an immunocompetent adult patient without risk factors for severe MSF.

**Conclusion:**

Myocarditis is an uncommon but severe complication of MSF. Clinicians should be aware of a possible cardiac involvement in patients with MSF. Close monitoring and an aggressive approach are essential to reduce mortality rates of MSF.

## Introduction

Mediterranean spotted fever (MSF) is a tick-borne acute febrile disease caused by *Rickettsia conorii*. The vector of the infection is the brown dog tick *Rhipicephalus sanguineus*, which is widespread in the Mediterranean area. MSF is typically characterized by fever, maculopapular rash and a black eschar at the site of the tick bite (‘tache noir’). Most cases follow a benign course, with a case fatality rate of 3–7 % among hospitalized patients (Duque *et al.*, 2012). Advanced age, chronic alcoholism, immunocompromised status, glucose-6-phosphate dehydrogenase deficiency, inappropriate antimicrobial therapy, delay in treatment, and diabetes are risk factors for severe MSF. Complications are described mainly in adult patients and include hepatic, renal, neurological and cardiac impairment (Colomba *et al.*, 2006, 2008). Among cardiac complications, pericarditis, myocarditis and heart rhythm disorders are uncommon complications in MSF and only a few cases have been reported in the literature (Ben Mansour *et al.*, 2014; Cascio *et al.*, 2011; Colomba *et al.*, 2008; de Groot *et al.*, 1984; Grand *et al.*, 1975; Lisa Catón, 1991). We describe a new case of acute myocarditis complicating MSF in an immunocompetent adult patient without risk factors for severe MSF.

## Case report

We describe the case of a 54-year-old man who was admitted to the infectious diseases clinic of the University Hospital of Palermo, Sicily, Italy in August 2014 because of MSF complicated by sepsis-induced multi-organ failure and myocarditis. He presented with fever for a few days. On admission, the patient was febrile, lethargic and with slurred speech, not oriented in space and time (GCS 11), with no signs of meningeal irritation. Vital signs were: pulse, 102 min^−1^; blood pressure, 80/50 mmHg; respiratory rate, 40 min^−1^. A diffuse maculopapular rash, involving the palms, was present. A dark crusted lesion, tache noire-like, was present on the left thigh. Diffuse wheezes were heard on pulmonary auscultation. Heart auscultation revealed paraphonic tones. The blood exam showed: white blood cell (WBC) 8.600 cells mm^−3^ (N 86.7 %, L 5.5 %), PLT 80.000 cells mm^−3^, AST/ALT 543/188 U l^−1^, creatinine 3.71 mg dl^−^^1^,and blood urea nitrogen (BUN) 66.8 mg dl^−1^; arterial blood gas analysis showed pH 7.39, pCO2 38.6 mmHg, pO2 41.9 mmHg, sO2 78.9 %, lactates 1.1 mmol l^−^^1^, bicarbonate 23.3 mmol l^−^^1^. We observed an increase in cardiac enzymes (peak of creatine kinase MJ3 fraction and troponin I of 800 UI l^−1^ and 2.52 mg ml^−1^, respectively). An electrocardiogram showed normal sinus rhythm with T-wave inversion in I, aVL, V4–V5–V6. An echocardiogram showed a dilated ventricle, reduced ejection fraction (35 %), and diffuse moderate hypokinesis. The cranial computed tomography (CT) scan was normal, the chest CT scan showed signs of severe emphysema, patchy interstitial infiltrate in the right lower lobe and mild pericardial effusion. Because of the multi-organ failure, the patient was transferred to the ICU, intubated, and given intravenous fluid therapy after the cranial CT results. Serological tests to detect *R. conorii* IgM and IgG [indirect immunofluorescence assay (IFI) and ELISA] were negative. *Rickettsia* PCR on blood was positive. Based on a presumptive diagnosis of MSF, the patient was promptly treated with chloramphenicol 3.5 g four times per day and ciprofloxacin 400 mg twice daily intravenously. A cardiology consultation suggested myocardial protection therapy with bisoprolol 1.25 mg orally once daily and ramipril 1.25 mg orally once daily. After two days the patient became afebrile, and after six days, the patient’s condition improved sufficiently that he was transferred to the Infectious disease unit. The *R. conorii* IFI and ELISA were repeated after one week, showing elevated IgM and IgG titers (IFI IgM–IgG 1/320–1/640; ELISA IgM–IgG 1/200–1/800). After six days of chloramphenicol, antibiotic therapy was switched to doxycycline 100 mg orally twice daily until the patient was discharged on hospital day 16. A follow-up electrocardiogram performed after two weeks demonstrated T-wave normalization. Echocardiogram findings after two weeks and one month were unchanged. At the six-month follow-up the patient was in good clinical condition.

## Discussion

Cardiac impairment is a rare complication of severe *Rickettsia *spp. infection. Myocarditis has been observed frequently at autopsy in fatal cases of Rocky Mountain spotted fever (Walker *et al.*, 1980; Bradford & Hackel 1978; Nilsson *et al.*, 2005). One case of Japanese spotted fever, one case of African tick-bite fever and very few cases of scrub typhus complicated with acute myocarditis have also been described (Fukuta *et al.*, 2007; Sittiwangkul *et al.*, 2008).

Regarding cardiac complication in MSF, several cases of pericarditis have been described. Few cases of myocarditis and very few cases of heart rhythm disorders have been reported. Only one case of coronary involvement has been described in Italy (Colomba *et al.*, 2008; Grand *et al.*, 1975). A scrupulous analysis of all publications resulted in five eligible articles describing five patients with cardiac involvement clearly related to *R. conorii* (Colomba *et al.*, 2008; Cascio *et al.*, 2011; Ben Mansour *et al.*, 2014; Salvi *et al.*, 1985). Data regarding clinical characteristics, therapy and outcome of these patients, along with our case, are analytically shown in Table 1. Cascio *et al*. (2011) describe the case of a 3-year-old boy with MSF who developed a transient right coronary artery ectasia. The authors suggest that it is more likely that coronary ectasia was the result of the rickettsial vasculitis. The inflammatory response to rickettsial infections triggered the cascade of events that led to Kawasaki syndrome (KS) (Cascio *et al.*, 2011). Considering the diagnosis of KS, treatment with intravenous immunoglobulin and aspirin was initiated while clarithromycin was continued to treat serologically confirmed MSF. Clarithromycin is considered one of the safest and most efficacious treatment (Cascio *et al.*, 2001, 2002). Among the only three cases of myocarditis related to *R. conorii* infection described in the literature, two were children and one a young adult (Ben Mansour *et al.*, 2014; Salvi *et al.*, 1985). Only in one case the diagnosis was performed with endomyocardial biopsy (Salvi *et al.*, 1985). The histological finding of myocarditis in the course of MSF is diffuse vasculitis with disruption of the vessel wall by a predominantly mononuclear-cell infiltrate. The target cell of rickettsiae is the vascular endothelial cell where it multiplies. The result is a widespread vasculitis of capillaries, arterioles and small arteries that correlates with the presence of *R. conorii* (Mekhloufi & Ait-Abbas, 2001).

**Table 1.** Clinical characteristics, therapy and outcome of patients with MSF and cardiac involvement

Authors, year, country	Sex/age (years)	Cardiac involvement	ECG	Echocardio	Serology	PCR	Biopsy	Therapy	Outcome
Colomba *et al.*, 2008, Italy	Male/40	Atrial fibrillation	AF	Normal	+	nd	nd	Doxycycline 100 mg bid for 7 days	Complete recovery
Cascio *et al.*, 2011, Italy	Male/3	Ectasia of coronary arteries	Normal	Dilated right coronary artery with hyperreflective walls	+	nd	nd	Intravenous immunoglobulin 2g kg^-1^ qd and clarithromycin 15 mg kg^-1^ qd for 14 days	Complete recovery
Ben Mansour *et al.*, 2014, Tunisia	Male/15	Myocarditis	ST segment elevation in anterior leads	Left ventricular dysfunction with 40 % ejection fraction	+	nd	nd	Vibramycine 200 mg qd and rifadine 10 mg kg^-1^ qd for 20 days	Complete recovery
Patil *et al.*, 2010, India	Male/1	Myocarditis	Not described	Dilated left ventricle with mild mitral and tricuspid regurgitation, reduced ejection fraction (25–30 %),	+	nd	nd	Doxycycline 100 mg bid (duration of therapy not indicated)	Complete recovery
Colomba, *et al.*, 2014, Italy	Male/54	Myocarditis	Normal sinus rhythm, T-wave inversion in I, aVL, V4-V5-V6	Dilated left ventricle, reduced ejection fraction (35 %), diffuse-moderate hypokinesis	+	+	nd	Chloramphenicol 3.5 g qid for 6 days, Doxycycline 100 mg bid for 10 days	Not recovered

PCR, Polymerase Chain Reaction; AF, atrial fibrillation; nd, no data available; bid, bis in die (twice daily); qd, quaque die (once daily); qid, quater in die (four times per day).

Even if a definitive diagnosis of myocarditis can be made only by endomyocardial biopsy, it is an invasive procedure that carries the risk of lethal complication and it is not recommended in the routine evaluation of patients with new-onset heart failure (Yancy *et al.*, 2013). Consequently, in our case we did not request endomyocardial biopsy to confirm the diagnosis. However, in our opinion, the clinical syndrome, cardiac biomarkers, and electrocardiographic and echocardiographic findings provided strong evidence of acute myocarditis. Early diagnosis and treatment allowed favourable evolution.

Among MSF cardiac complications, arrhythmia has been rarely reported. Inflammation may play a role in the pathogenesis of atrial fibrillation. Inflammatory cells infiltrating the left atrial endocardium have been demonstrated in patients affected by this arrhythmia. Moreover, the pulmonary veins have a crucial role as one of the key trigger sites for the onset of atrial fibrillation. *R. conorii* could trigger atrial fibrillation because of its ability to invade endothelial cells and cause perivascular inflammation with activation of the acute phase response (Colomba *et al.*, 2008). Besides a case we described (Colomba et al., 2008), another case of atrial fibrillation and one case of supraventricular tachycardia in a child have been described (Scaffidi *et al.*, 1981; de Groot *et al.*, 1984).

Myocarditis is an uncommon but severe complication of MSF. Clinicians should be aware of a possible cardiac involvement in patients with MSF. Close monitoring and an aggressive approach are essential to reduce mortality rates of MSF.

## References

[R1] Bellini C. Monti M. Potin M. Ave A. D. Bille J. Greub G.(2005). Cardiac involvement in a patient with clinical and serological evidence of African tick-bite fever. BMC Infect Dis 5 90. 10.1186/1471-2334-5-90 16242016 PMC1274315

[R2] Ben Mansour N. Barakett N. Hajlaoui N. Haggui T. Filali T. Dahmen R. Fehri W. Haouala H.(2014). Acute myocarditis complicating Mediterranean spotted fever. A case report. Ann Cardiol Angeiol 63 55–57. 10.1016/j.ancard.2011.05.003 21664598

[R3] Bradford W. D. Hackel D. B.(1978). Myocardial involvement in Rocky Mountain spotted fever. Arch Pathol Lab Med 102 357–359. 580868

[R4] Cascio A. Colomba C. Antinori S. Paterson D. L. Titone L.(2001). Clarithromycin versus azithromycin in the treatment of mediterranean spotted fever in children: a randomized controlled trial. Clin Infect Dis 33 409–411. 10.1086/321864 11740701

[R5] Cascio A. Colomba C. Antinori S. Paterson D. L. Titone L.(2002). Clarithromycin versus azithromycin in the treatment of Mediterranean spotted fever in children: a randomized controlled trial. Clin Infect Dis 34 154–158. 10.1086/338068 11740701

[R6] Cascio A. Maggio M. C. Cardella F. Zangara V. Accomando S. Costa A. Iaria C. Mansueto P. Giordano S.(2011). Coronary involvement in Mediterranean spotted fever. New Microbiol 34 421–424. 22143818

[R7] Colomba C. Saporito L. Polara V. F. Rubino R. Titone L.(2006). Mediterranean spotted fever: clinical and laboratory characteristics of 415 Sicilian children. BMC Infect Dis 60. 10.1186/1471-2334-6-60 PMC1435909 16553943

[R8] Colomba C. Saporito L. Colletti P. Mazzola G. Rubino R. Pampinella D. Titone L.(2008). Atrial fibrillation in Mediterranean spotted fever. J Med Microbiol 57 1424–1426. 10.1099/jmm.0.2008/002162-0 18927423

[R9] Colomba C. Imburgia C. Trizzino M. Titone L.(2014). First case of Mediterranean spotted fever-associated rhabdomyolysis leading to fatal acute renal failure and encephalitis. Int J Infect Dis 26 12–13. 24846600 10.1016/j.ijid.2014.01.024

[R25] de Groot R. Oranje A. P. van der Heyden A. J. Vuzevski V. D. Schaap G. J. van Joost T.(1984). *Rickettsia conorii* infection complicated by supraventricular tachycardia in a ten year-old child. Acta Leiden 52 45–52. 6536143

[R10] Duque V. Ventura C. Seixas D. Barai A. Mendonca N. Martins J. da Cunha S. Melico-Silvestre A.(2012). Mediterranean spotted fever and encephalitis: a case report and review of the literature. J Infect Chemother 18 105–108. 21879306 10.1007/s10156-011-0295-1

[R11] Fukuta Y. Mahara F. Nakatsu T. Yoshida T. Nishimura M.(2007). A case of Japanese spotted fever complicated with acute myocarditis. Jpn J Infect Dis 60 59–61. 17314430

[R12] Grand A. Guillermet F. N. Despret P. Kreuter M. Hintzy L.(1975). Acute pericarditis caused by *Rickettsia conori*. Arch Mal Coeur Vaiss 68 1225–1231. 816289

[R13] Lisa Catón V. Ochoa Gómez F. J. Peña Somovilla J. L. Salcedo Aguilar J. Ruiz Valverde A. Campo Hernández J. M.(1991). Myocarditis caused by Mediterranean boutonneuse fever. Enferm 9 59. 2029562

[R14] Lunge S. B. Patil V. Ambar S. Naik V.(2015). Malignant Mediterranean spotted fever. Indian Dermatol Online J 6 s1–4. 10.4103/2229-5178.171050 26904440 PMC4738506

[R15] Marcon G. Callegari E. Scevola M. Bettin M. Pozzati G. Zolli M. Carlassara G. B.(1988). Acute rickettsial myocarditis. Description of a clinical case and review of the literature. G Ital Cardiol 18 72–75. 2968291

[R16] Mekhloufi E. Y. Ait-Abbas N.(2001). [Mediterranean spotted fever complicated by myocarditis. Presse Med 30 586. 11317917

[R17] Nilsson K. Liu A. Påhlson C. Lindquist O.(2005). Demonstration of intracellular microorganisms (*Rickettsia* spp., *Chlamydia pneumoniae*, *Bartonella* spp.) in pathological human aortic valves by PCR. J Infect 50 46–52. 10.1016/j.jinf.2003.10.009 15603840

[R18] Patil H. Shahapur P. Bidari L. H.(2010). Myocarditis and* Rickettsia conorii *infection. PediatricOncall [serial online].

[R19] Salvi A. Della Grazia E. Silvestri F. Camerini F.(1985). Acute rickettsial myocarditis and advanced atrioventricular block: diagnosis and treatment aided by endomyocardial biopsy. Int J Cardiol 7 405–409. 3838736 10.1016/0167-5273(85)90094-4

[R20] Saporito L. Giammanco G. M. Rubino R. Ingrassia D. Spicola D. Titone L. Colomba C.(2010). Severe Mediterranean spotted fever complicated by acute renal failure and herpetic oesophagitis. J Med Microbiol 59 990–992. 10.1099/jmm.0.015891-0 20508004

[R21] Scaffidi A. Furitano G. Scaffidi L.(1981). Cardiovascular involvement and complications in Mediterranean boutonneuse fever. Minerva Med 72 2097–2108. 7254649

[R22] Sittiwangkul R. Pongprot Y. Silviliarat S. Oberdorfer P. Jittamala P. Sirisanthana V.(2008). Acute fulminant myocarditis in scrub typhus. Ann Trop Paediatr 28 149–154. 10.1179/146532808X302189 18510826

[R23] Walker D. H. Paletta C. E. Cain B. G.(1980). Pathogenesis of myocarditis in Rocky Mountain spotted fever. Arch Pathol Lab Med 104 171–174. 6767463

[R24] Yancy C. W. Jessup M. Bozkurt B. Butler J. Casey D. E. Drazner M. H. Fonarow G. C. Geraci S. A. Horwich T.(2013). ACCF/AHA guideline for the management of heart failure: a report of the American College of Cardiology Foundation/American Heart Association Task Force on Practice Guidelines. J Am Coll Cardiol 62 e147–239. 10.1016/j.jacc.2013.05.019 23747642

